# The Stepping Stone Clubhouse Evaluation: Exploring Members’ Experiences, Service Engagement, and Perceived Impact of the Clubhouse International Model

**DOI:** 10.1007/s10597-024-01397-x

**Published:** 2025-01-16

**Authors:** Brianna S. Fjeldsoe, Kathryn Vitangcol, Tayla Lamerton, Melanie Sennett, Daniel Helton, Fotini Hardy, Marianne Wyder, Zoe Cunningham, Martina O. McGrath, Morag Roseby, Andrew McLean, Scott Brown, Sheleigh Lawler

**Affiliations:** 1 Enable Health Consulting, The University of Queensland (School of Public Health (Adjunct)), Brisbane, Queensland Australia; 2 Enable Health Consulting, Brisbane, Queensland Australia; 3 Stepping Stone Clubhouse, Brisbane, Queensland Australia; 4 Metro South Addiction and Mental Health Services, Stepping Stone Clubhouse Management Committee, Brisbane, Queensland Australia; 5 Queensland Mental Health Commission, Brisbane, Queensland Australia; 6 School of Public Health, The Unversity of Queensland, Brisbane, Queensland Australia

**Keywords:** Psychosocial rehabilitation, Clubhouse model, Mental health, Mental illness, Community services

## Abstract

The Clubhouse Model of Psychosocial Rehabilitation provides non-clinical social support for adults living with a diagnosed mental illness or self-reported mental ill-health (referred to as ‘members’). The Stepping Stone Clubhouse in Brisbane, Australia was evaluated between August 2022 and August 2023 using a participatory action research approach. Data was sourced from member surveys, member interviews, and an existing Clubhouse Member Database. Outcomes included members’ self-reported psychosocial recovery, social connectedness, quality of life, frequency of hospitalizations, and their attainment of employment and/or education aspirations. In this cross-sectional evaluation, it was hypothesized that *existing members* (membership: 11 months – 28 years) would report better outcomes than *new members* (membership: 0–14 days). In total, 161 existing members and 76 new members completed a survey. Twenty-three members also participated in a semi-structured interview. Participants were aged on average 47.1 years (± 13.5), 62% were male and 31% had a primary diagnosis of schizophrenia/schizoaffective disorder. Existing members reported better scores than new members for: three of the four psychosocial recovery domains (Functional Recovery: 78.0% vs. 74.0%, *p* = 0.01; Symptom Management Recovery 69.5% vs. 65.2%, *p* = 0.03; Social Recovery 74.3% vs. 70.0%, *p* = 0.01); social connection with other members (38.1 vs. 32.2, *p* = 0.03) and staff (44.5 vs. 38.1, *p* = 0.02); quality-of-life summary scores (4.6 vs. 4.1, *p* = 0.01), and rates of mental health-related hospitalization (16% vs. 41%, *p* < 0.01). Existing members were also more likely to be in some form of employment, compared to new members (*p* = 0.01). There were no significant differences between existing and new members for their educational goals, with 58% of each group wanting further formal education. Stepping Stone members have better outcomes than members who have recently joined the Clubhouse. This evaluation was successful because it intentionally built evaluative capacity and empowered member-centric processes.

## Introduction

The Clubhouse Model of Psychosocial Rehabilitation (the Clubhouse Model) provides intentionally formed, non-clinical social support for adults living with a diagnosed mental illness or self-reported mental ill-health (referred to as ‘members’) (Propst, 1997). The Clubhouse Model creates social support through therapeutic working communities composed of members and staff who work collaboratively to engage in Clubhouse activities. A core tenet of the Model is the belief that every member possesses the capacity to recover sufficiently from the effects of mental illness, to lead a personally satisfying life. A tenet actively realized through harnessing the restorative power of work, and work-mediated relationships. The Clubhouse Model fosters member involvement in all organizational operations, nurturing individual strengths through diverse programs encompassing employment and education support, healthy active living, social connection and recreation, and comprehensive community and individual supports to aid wellbeing. A distinctive driver of the Clubhouse Model is the mutual reciprocity between the Clubhouse and its members (Coniglio et al., 2012). This relationship thrives on voluntary participation without time limits or commitments and unconditional re-engagement with programs following an absence. Membership signifies belonging to a community with a collective responsibility for the success of the Clubhouse.

Currently, there are over 350 Clubhouses in 32 countries around the world. Previous evaluations of the Clubhouse Model have confirmed its positive effects on adults diagnosed with mental illness (McKay et al., 2018; Propst, 1997; Hinchey et al., 2023). These evaluations have predominantly measured outcomes related to employment, quality of life, social relationships and hospitalization related to mental health (McKay et al., 2018). A systematic review of 52 Clubhouse evaluations concluded that the Clubhouse Model has sufficient evidence to assure administrators of its effectiveness as one component of a spectrum of rehabilitative services for persons living with mental illness (McKay et al., 2018). In particular, the Clubhouse Model has been identified as effectively supporting members to obtain and maintain employment for extended periods compared to other vocational rehabilitation programs (Schonebaum et al., 2006; Macias et al., 2006; Meyer et al., 2023). However, to date, few Clubhouse evaluations have comprehensively measured short-term through to long-term impacts for member’s psychosocial recovery, social connectedness, quality of life, frequency of hospitalizations, and their attainment of employment and/or education aspirations. No publications have evaluated an Australian-based Clubhouse and there is also limited literature on how the Clubhouse Model supports capacity for evaluative practice in everyday operations.

Stepping Stone Clubhouse is an Australian-based community organization that was established in 1994. It is accredited against the International Clubhouse Model and is one of the twelve international training bases, providing training to Clubhouses around the world and the only training base in the Southern hemisphere. In 2024, there were six Clubhouses in Australia (only two accredited) and of those Stepping Stone was the largest and longest running. Between August 2022 and August 2023, Stepping Stone Clubhouse undertook a comprehensive evaluation project that aimed to: (1) capture its impact on members’ mental health and wellbeing, and social and civic participation; and, (2) build the Clubhouse’s capacity to integrate monitoring and evaluation practices into its culture and practice.

This article describes the overall findings from the Stepping Stone Clubhouse evaluation project, including members’ self-reported impacts of Stepping Stone on their psychosocial recovery, social connectedness, quality of life, frequency of hospitalizations, and their attainment of employment and/or education aspirations. In this cross-sectional evaluation, it was hypothesized that *existing members* (membership: 11 months – 28 years) would report better outcomes than *new members* (membership: 0–14 days). This article also reflects on the evaluation capacity building efforts and member-centric processes employed during the evaluation project.

## Methods

### Study Design

This study employed a participatory action research (PAR) approach with mixed methods data collection to comprehensively examine the impacts of Stepping Stone Clubhouse on members. The outcomes for the study were collaboratively defined by the Evaluation Reference Group, which was made up of the Evaluation Reference Group. This Evaluation Reference Group collaboratively created a logic model to define the short, medium, and longer-term outcomes experienced by members and, importantly, the assumptions in how these outcomes were generated through Clubhouse membership. The Evaluation Reference Group was the governing body for this study including strategic oversight, study design selection, survey design, reflective refinement practices throughout and ultimate interpretation of data analysis.

Data for this evaluation was sourced from (1) member surveys, (2) member interviews, and (3) an existing Clubhouse Member Database. This study included cross-sectional surveys from members. Since completing this cross-sectional data collection, the Evaluation Reference Group are embedding data collection processes to longitudinally track the outcomes of new members as they progress. However, this data has not yet been collected.

### Participants and Sampling

Participants in this study were recruited from the membership population of the Stepping Stone Clubhouse (adults aged over 18 with a self-reported primary mental illness) using simple random sampling. Participants were recruited as either a *“new”* or an *“existing”* member. New members joined during the evaluation period (August 2022 - August 2023) and were all invited to complete the member survey between zero and 14 days of having joined. Existing members were all people who were members before August 2022 who had consented to receiving contact from Stepping Stone Clubhouse and either had an active email address or attended the Clubhouse during a data collection wave (described below). The study was designed to have 95% power with α = 0.05 to compare outcomes between two groups of members, resulting in a minimum target sample size of at least 120 participants (60 in each group) using a priori power analysis.

### Recruitment

Recruitment of existing members took place in two waves (August 2022, January 2023), with each data collection wave lasting two weeks. Various recruitment strategies were employed to optimize member participation throughout the study. Firstly, an email invitation was disseminated to all eligible members with a direct link to the members survey. Participants were invited to complete the survey in their own time online, or attend the Clubhouse and complete the survey, with the option to be supported to complete the survey by one of the evaluation consultants (interview-administered). Additionally, during each two-week data collection wave staff and members spoke to other members about the evaluation and invited them to speak to one of the evaluation consultants in-person. Finally, noticeboards within the Clubhouse facility and pre-existing Stepping Stone Clubhouse social media channels were used to promote the evaluation project. No members were actively approached by the evaluation consultants to participate in the evaluation.

Recruitment of new members happened during the face-to-face Orientation session that is conducted by members and staff of Stepping Stone Clubhouse. This Orientation session marks the point of a person transitioning from an interested individual to a Clubhouse member. At this Orientation session, the evaluation was introduced, and new members were informed of their right to not participate, to remain anonymous, and their right to withdraw at any time without consequences.

Participants expressed consent through diverse means, with consideration given to accommodating changes in participation preferences across the data waves. For surveys, members provided written consent if completing the survey independently or verbal consent for interview-administered surveys. Through the survey, participants also provided written permission to link their survey data to service engagement data through their unique database identifier. In interviews, members provided written and/or verbal consent, with the option for audio-recording.

### Ethics

This project was reviewed by The University of Queensland’s Human Research Ethics Committee. It received ethical clearance in August 2022. The paid evaluation consultants (BF, TL, KV) had no personal affiliations with the Clubhouse. The ethics and quality of the study process were maintained and monitored via regular meetings of the Evaluation Reference Group and the provision of feedback to the evaluators, with emphasis on reflexivity (Probst, 2015).

### Data Sources and Measures

Data for this evaluation was sourced from (1) member surveys, (2) member interviews, and (3) an existing Clubhouse Member Database. Member surveys were administered to both new and existing members, through either electronic (via SurveyMonkey™) or paper-based formats. Participants had the option of completing the surveys via an interview-administered format or via self-administration. One-to-one semi-structured interviews were also conducted for interested participants. Pre-existing data collected by the Clubhouse was accessed via the Member Database, which provided demographic information (e.g., age, gender, self-reported diagnosis, and length of membership) and engagement data for each member. This pre-existing data was only linked to member surveys if the member provided informed, written consent for the linkage.

### Evaluation Indicators

#### Engagement Measures

Engagement measures were accessed through the Member Database and included the number of occurrences and duration of Stepping Stone services, including:

- *Work-Ordered Day (WOD)*: the Clubhouse employs a “work-ordered day” model (Beard et al., 1982), where members and staff work side-by-side on the tasks and roles needed to run the Clubhouse. Members work in units like clerical, hospitality, reception and maintenance, education and employment, which promotes their vocational skills and purpose.
- *Individual Support & Employment Support (IS & ES)*: IS provides one-on-one assistance, addressing members’ personal and recovery-related goals, while ES aids in goal-setting, building employment and/or education skillsets, and securing community employment and/or education aligning with members’ skills and aspirations.
- *Transitional Employment (TE)*: services encompass job development, and supported employment initiatives, focusing on individualized placements (Stepping Stone Clubhouse typically offers 12–15 different TE roles), career development, and fostering members’ integration into competitive employment for independence and overall well-being.
- *Outreach*: contacts are initiated by staff or members through various channels (e.g., telephone calls, text messaging, instant messaging platforms, emails) to members to offer support, check-in, and inform them of upcoming opportunities. Outreach is conducted for members when their Clubhouse attendance pattern changes, when it is their birthday and during hospitalizations.
- *Social Recreation*: organized social and/or recreational activities to enhance social connections and provide leisure opportunities for members during evenings, weekends, and public holidays.
- *Support Coordination and Psychosocial Recovery Coaching (SC & PRC)*: coaching that occurs between a member and staff on holistic recovery, fostering resilience, and wellbeing across clinical, social, vocational, and personal dimension. This coaching also assists members in understanding and navigating the National Disability Insurance Scheme (NDIS: a scheme that provides funding to eligible people with disability in Australia).

#### Outcome Measures

Outcomes for this evaluation were measured through the member survey and included:

- *Psychosocial recovery* was measured using The Recovery Assessment Scale - Domain and Stages (RAS-DS), which is a 38-item tool designed to assess and measure the recovery of individuals in mental health recovery (Hancock et al., 2015). On a 4-point Likert scale, participants respond to statements related to their personal recovery (looking forward), functional recovery (doing things I value), symptom management (mastering my illness) and social recovery (connecting and belonging). Higher scores indicate better recovery, and a score: below 50% represents an area for work, between 50 and 75% represents a transitional area and above 75% represents an area of success. The tool has been shown to be acceptable among adults with mental illness, valid compared to other measures and sensitive to detecting change over time (Hancock et al., 2015; Scanlan et al., 2018).
- *Social connectedness* was measured using the 12-item Client Interaction Scale (CIS), which separated out connection with other members at the Clubhouse from connection with the Clubhouse staff (Brekke & Aisley, 1990). Rather than seeking an objective assessment of the Clubhouse environment, the CIS attempts to measure the clients’ self-perceived level of integration into the client-to-client environment. For this study, the term *client* was changed to *member* to be consistent with the context of the Clubhouse environment. In this scale, higher scores reflect stronger social connections.
- *Quality of life* was measured using the Manchester Short Assessment of Quality of Life (MANSA) (Priebe et al., 1999). From this 7-point Likert scale (1 (couldn’t be worse) to 7 (couldn’t be better), an overall satisfaction score was calculated across, where higher scores indicate greater satisfaction with life. The MANSA was specifically developed with adults living with serious mental illness and comprises of two distinct but correlated factors examining satisfaction with: (1) life and health related aspects (such as satisfaction with life as a whole, physical and mental health, leisure activities and friends, job/unemployment and financial situation, and sex life); (2) living environment (family, accommodation, safety, and their living situation) (Petkari et al., 2020).
- *Mental-health related hospitalizations* was measured with a bespoke two-item question, which required members to answer whether they had been to hospital in the past 6 months for their mental health, and if so what type of hospitalization (called emergency services, presented to emergency department and/or admitted to hospital).
- *Employment and education aspirations* were measured with bespoke questions, which asked members to select from closed-response options their current employment status and their aspirations for employment. Members were also asked to select their highest attained education, as well as their aspirations for further education.

#### Semi-Structured Interviews

The semi-structured interviews in this project aimed to understand members’ experiences of Stepping Stone and what they viewed to be the most significant impacts on their life. Members were able to opt-in to a semi-structured interview via a question in the survey at Wave One or Two. The interviews were conducted by one of the evaluation consultants (BF, TL, KV, who identify as female and as not living with a mental illness), one-on-one with a member (or with a support staff present as requested by the member), in a quiet space at the Clubhouse, or via videoconference, and were audio-recorded. Before the interview, participants were given a brief list of the topics to be discussed, during the interview they were verbally reminded that answering questions was voluntary and that their stories would remain confidential. After the interviews, participants were able to request a debrief with a chosen Stepping Stone staff member if desired. The interview guide included prompts aimed at understanding how participants constructed meaning of their experiences of “recovery in the Clubhouse context”.

### Data Analysis

Qualitative data underwent thematic analysis conducted by two independent authors (TL, BF). These authors individually coded all interview recordings and/or written scripts for meaning in Microsoft Word™. Collaboratively, they discussed differences and similarities, building consensus on descriptive codes and uncovering latent interpretations, ultimately identifying themes within and across the data. This iterative analysis aimed to provide a nuanced understanding of the Clubhouse experience. The themes were presented to the Evaluation Reference Group and refined for clarity and language.

Quantitative data underwent cleaning and analysis using Microsoft Excel and R version 4.3.1. Cross-sectional comparisons involved initial descriptive statistics, followed by bivariate analysis using chi-squared tests and t-tests. The statistical significance of these tests was set at *p* < 0.05.

Two interim reflective processes were conducted by the Evaluation Reference Group at two time points before the completion of the study, whilst data collection was ongoing. These sessions were an important aspect of the PAR approach, and enabled the Group to critique recruitment processes and data collection processes, explore missing data patterns in the survey and refine study protocols as required. In addition, the paid evaluation consultants presented these interim reflections to a broader group of Stepping Stone members to gather further insights and feedback on study processes.

### Participants

There were 500 members eligible to participate as an existing member in August 2022. Between August 2022 and August 2023, 135 members joined Stepping Stone Clubhouse and were therefore eligible to participate in this evaluation as a new member. In total, 237 participants completed a member survey 161 existing members (32% of eligible participants invited by email) and 76 new members (56% of eligible participants). There was a relatively even distribution of participants who opted to complete an interview-administration of the member survey (57%) and a self-administered emailed link of the survey (43%). One survey respondent opted to complete the paper-based version of the survey. The majority of participants (87%, 68 new members and 138 existing members) gave permission to link their survey data with their demographic and engagement data from the Member Database. Twenty-three members completed a semi-structured interview at either Wave One or Two. The sample recruitment targets for the cross-sectional survey were exceeded for both groups and the sample of members engaging in semi-structured interviews led to saturation of themes.

Demographic characteristics of participants are reported in Table 1. The mean age of the participants was 47.1 (± 13.5), 62% were male and 31% had a self-reported primary diagnosis of schizophrenia/ schizoaffective disorder. The demographic characteristics of the evaluation sample were representative of the broader Clubhouse membership (data not shown). However, there were meaningful and statistically significant differences in the demographic characteristics of the existing members compared to the new member samples (Table 1). New members were younger, and less likely to have a diagnosis of schizophrenia/schizoaffective disorder (Table 1). Both new and existing members were similar in the distribution of gender across samples (majority male).

**Table 1:** Demographic and mental health characteristics of clubhouse members who participated in the evaluation

	Evaluation sample (*n* = 237)	Existing members (*n* = 161)	New members (*n* = 76)	*p*-value
Age, mean (SD)	47.1 (13.5) *n* = 224	49.7 (12.2) *n* = 159	39.7 (13.3) *n* = 65	< 0.01
Length of membership at time of survey completion, median (SD)	6.4 years (7.4) *n* = 230	8.0 years (7.2) *n* = 157	0 days (3.9) *n* = 73	< 0.01
Gender, n (%) Male Female Non-Binary/Transgender	142 (62) 81 (35) 6 (3)	97 (61) 60 (38) 2 (1)	45 (64) 21 (30) 4 (6)	0.18
Primary Mental Illness Diagnosis, n (%)* Depression Other affective problems Anxiety disorders Other anxiety-related problems Schizophrenia/schizoaffective disorder Other mental & behavioral problems Other psychoses	58 (26) 35 (16) 20 (9) 9 (4) 69 (31) 24 (11) 7 (3)	39 (26) 29 (19) 13 (9) 3 (2) 55 (37) 7 (5) 4 (3)	19 (26) 6 (8) 7 (10) 6 (8) 14 (19) 17 (24) 3 (4)	0.01

*Diagnosis was available for *n* = 150 of existing members and *n* = 72 of new members

### Member Engagement with Stepping Stone Clubhouse

Almost all participants (99%, *n* = 234) who completed a member survey engaged with some form of activity offered at Stepping Stone Clubhouse. Table 2 shows the proportion of participants who accessed each Clubhouse activity type at least once over the 12-month study period. Outreach was the most commonly experienced Clubhouse activity by participants, with the occurrence of remaining activities varying for new and existing members. Compared to existing members, a higher proportion of new members accessed Work-Ordered Day, and a lower proportion accessed Social Recreation, Support Co-ordination and Psychosocial Recovery Coaching.

**Table 2:** Proportion of participants who had experienced at least one occurrence of each activity type in the 12-month evaluation period

Type of Clubhouse Activity	Existing members (*n* = 160)	New members (*n* = 74)
Outreach	97%	88%
Work-Ordered Day	36%	68%
Individual and Employment Support	43%	41%
Social Recreation	47%	22%
Support Co-ordination and Psychosocial Recovery Coaching	39%	4%

When examining the volume of services accessed as a proportion of total occurrences (Table 3), new members most frequently accessed Work-Ordered Day (38.0%) followed by Individual and Employment Support (21.1%; Table 3). Existing members most frequently accessed Support Co-ordination and Psychosocial Recovery Coaching (44.4%) and Individual and Employment Support (19.1%; Table 3).

**Table 3:** Volume of engagement as a proportion of total number of services and supports accessed across all service types

Type of Clubhouse Activity	Existing members (*n* = 160)	New members (*n* = 74)
Outreach	13%	20%
Work-Ordered Day	18%	38%
Individual and Employment Support	19%	21%
Social Recreation	6%	7%
Support Co-ordination and Psychosocial Recovery Coaching	44%	14%

However, the patterns of service engagement shift again when considering the duration of each activity (i.e., in hours/occurrence). Although Outreach had a high number of total occurrences for both new and existing members, the time spent receiving Outreach is very small (0.9%) in comparison to the other activities. When considering time spent accessing an activity (hours/occurrence), existing and new members most frequently accessed Work-Ordered Day, but for new members a Work-Ordered Day accounted for a higher proportion of their time (67.9% of time) than it did for existing members (38.6% of time).

### Qualitative Findings about Member Engagement

The Stepping Stone journey is not linear, nor is it one size fits all - it is unique to the needs of the member. Members expressed a feeling of being supported (not pressured) to progress. This individualized journey is supported well by the flexibility of the Clubhouse Model and is created by the diversity of opportunities (employment, education, work-ordered day, social recreation, recovery coaching, housing, service navigation). Members found that because they could access services differently than their peers and also differently over time, they could get the help they needed no matter where they were in their journey. On a longer-term scale, members talked about stepping away from the Clubhouse and then stepping back in with no judgment. Having a membership without time-limits and the ability to step in and out as needed is deeply valued by members.

“I could take three months off and not come in here if I needed to get over an injury. I could come in on that first day after not being here for three months and it’s like I never left. I’d be just as welcomed. Just as valued” – No age provided, male, member for 1 year.

Another key finding of the qualitative interviews was that Outreach created a sense of belonging, even when members were not at the physical Clubhouse. An Outreach was also noted as the catalyst for bringing members back into the Clubhouse when they were struggling. Members spoke about feeling *“in the dark”* or *“feeling low”* and that a Clubhouse Outreach call or message was a lifeline that assisted them through those challenging times.

“Even though I don’t come here very often, I still feel a part of Stepping Stone. Especially during COVID, they would send a daily text, and my support worker would still reach out and say, have you heard of this or that coming up? I came back a couple of weeks ago but I forgot how much I love it here so I’m planning on coming back in more often.” – 31-year-old female, member for 5 years.

### Outcomes on Members from Stepping Stone Clubhouse

#### Psychosocial Recovery

Psychosocial recovery scores were significantly higher for existing members compared to new members across all domains except Personal Recovery (Table 4).

**Table 4:** Means and standard deviations (SD) of outcome scores for existing and new *members*

	Existing members (*n* = 157) mean (± SD)	New members (*n* = 75) mean (± SD)	*p*-value difference b/w groups
Psychosocial Recovery scores			
Personal Recovery	73.3% (± 14.2)	71.6% (± 16.0)	0.11
Functional Recovery	78.0% (± 14.2)	74.0% (± 16.3)	0.01
Symptom Management Recovery	69.5% (± 17.0)	65.2% (± 19.5)	0.03
Social Recovery	74.3% (± 17.0	70.0% (± 17.6)	0.01
Social connectedness scores			
Connectedness with other members	38.1 (± 16.8)	32.2 (± 20.1)	0.03
Connectedness with staff	44.5 (± 17.3)	38.1 (± 20.8)	0.02
Quality of Life scores			
Quality of life (QoL) - Summary score	4.6 (± 1.2)	4.1 (± 0.9)	< 0.01
QoL item – financial situation	4.1 (± 1.7)	3.3 (± 1.6)	< 0.01
QoL item – job OR unemployment	4.8 (± 1.5)	3.6 (± 1.5)	< 0.01
QoL item – mental health	4.4 (± 1.6)	3.8 (± 1.4)	< 0.01
QoL item – physical health	4.3 (± 1.6)	3.8 (± 1.5)	0.06
QoL item – sex life	3.6 (± 2.0)	3.9 (± 1.9)	0.32
QoL item – life as a whole	4.6 (± 1.4)	3.9 (± 1.4)	< 0.01
QoL item – leisure activities	4.5 (± 1.5)	4.0 (± 1.5)	0.02
QoL item – friendships	4.4 (± 1.6)	4.0 (± 1.6)	0.15
QoL item – relationships with your family	4.8 (± 1.6)	4.3 (± 1.7)	0.06
QoL item – accommodation	5.0 (± 1.7)	4.5 (± 1.8)	0.06
QoL item – the people that you live with OR living alone	5.1 (± 1.5)	4.8 (± 1.7)	0.14
QoL item – personal safety	5.2 (± 1.4)	4.9 (± 1.5)	0.17
Hospitalizations	Existing (*n* = 161) n (%)	New (*n* = 76) n (%)	p-value difference b/w groups
Hospitalized Not hospitalized Prefer not to say	26 (16) 131 (81) 4 (2)	31 (41) 41 (54) 4 (5)	X^2^ = 18.5, *p* < 0.01

#### Social Connectedness

Existing members scored higher than new members in social connections with both other members and staff, and the cross-sectional comparisons were statistically significantly higher scores (*p* = 0.03 and *p* = 0.02, respectively; Table 4).

#### Quality of Life

Existing members also had higher quality-of-life summary scores (4.6 ± 1.2) than new members (4.1 ± 0.9), and this cross-sectional comparison was statistically significant (*p* = 0.01; Table 4). Existing members were closer to feeling “mostly satisfied” with their quality of life, and new members on average report feeling “mixed”. Both new and existing members were most satisfied with their personal safety (Table 4). New members were least satisfied with their financial situation and existing members were least satisfied with their sex life. Existing members were significantly more satisfied than new members for: life as a whole today (*p* < 0.01), leisure activities (*p* = 0.02), mental health (*p* < 0.01), employment status (*p* < 0.01), and financial situation (*p* < 0.01; Table 4). These domain-specific findings align with the areas of life where Stepping Stone is best positioned to make a difference through the services and supports on offer.

#### Mental Health-Related Hospitalizations

New members had significantly higher rates of mental health-related hospitalization (41%, 31 out of 76 members) than existing members (16%, 26 out of 161 members) and this difference was statistically significant (*p* < 0.01; Table 4).

#### Employment and Education Outcomes

Members’ current employment status was statistically different between new and existing members, where existing members were more likely to be in some form of employment, compared to new members (Table 5). In addition, of the 160 existing members, 44.3% had a goal of maintaining or acquiring some form of employment, and 7.4% would like to participate in the Work-Ordered Day. Of the 74 new members, 58.9% had a goal of maintaining or acquiring some form of employment, and 6.5% reported they would like to have the Work-Ordered Day.

**Table 5:** Employment and education statuses and aspirations for existing and new members

	New Members (*n* = 74)	Existing Members (*n* = 160)	Group difference *p*-value
*Current Employment Status*			
Form of employment*	13.0%	24.5%	0.01
Work-ordered day	0%	13.3%	
Unemployed	44.7%	14.6%	
*Employment Aspirations*			
Form of employment*	58.9%	44.3%	0.61
Work-ordered day	6.9%	7.4%	
*Current Education Status*			
Did not finish high school	26.6%	15.4%	0.09
Complete high school	14.7%	15.5%	
Certificate/Diploma	33.3%	29.5%	
Tertiary qualifications	24.0%	36.6%	
*Education Aspirations*			
Do not want further education	25.3%	37.8%	0.17
Want further education**	58.7%	58.1%	

*Includes independent, supported, and transitional employment

**Includes completing high school and acquiring post-secondary^ or tertiary-level† education

^Post-secondary education includes Certificate II/III/IV

†Tertiary-level education includes Diploma/Advance diploma, Bachelor’s degree, Graduate Certificate/Graduate Diploma, and Postgraduate degree

From the qualitative interviews, a core experience of being a member is that *“you are safe”.* Safety is created in many ways, including the safety to enter the workforce (through the Work-Ordered Day or transitional employment (TE)). Members talked about how they felt protected by Stepping Stone in their transitional employment (TE), which was not what they had experienced in previous independent employment.

“I can go for my goals a safe bit at a time, just a bit at a time…with the TE there are safe jobs where staff can be around you, so I think it’s just allowed me to go out of my comfort zone and work through day to day things that I struggle with… It’s a guaranteed place of safety. Especially for myself and others out there working, feeling safe is really important” – 32-year-old male, member for 7 years.

Of the 160 existing members, 58.1% expressed wanting further formal education of some sort, whereas for new members (*n* = 74), 58.7% expressed wanting further formal education of some sort (Table 5). There were no statistically significant differences between existing and new members for their current education level or aspirational educational goals.

#### Qualitative Findings about Impacts on Members

When members spoke about the impacts of Stepping Stone Clubhouse on their lives, they centered around two main themes: the magnitude or depth of the impacts experienced and feeling accepted and valued. Many members described how Stepping Stone Clubhouse had *“changed their lives”*, and for some, *“saved their lives”*. Some members became overwhelmed by emotion when describing the impact that Stepping Stone Clubhouse has had on their lives. The impacts that members spoke about ranged from suicide prevention, to reducing hospitalization, to engaging in meaningful work, to learning new skills, to being needed or recognized in a community space. Regardless of the nature of the impact, members commonly referred to the immense depth of how Stepping Stone had impacted their lives.

“Ended up coming back in [to Stepping Stone Clubhouse], and bit by bit went from driving around with the means of suicide, to not having it six months later, then about four years later I done something that I thought I’d never ever do in my life, and I went back to work.” – 68-year-old male, member for 9 years.

“You know coming to Stepping Stone you can just be yourself and I guess that’s now resonating into other aspects of my life……. since coming to Stepping Stone, that’s when everything started changing.” - No age provided, male, member for 4 years.

“It has transitioned me from being in a position where I was never going to work, never have any sort of independence, to basically getting work, finding my feet basically, and knowing that they’re always there…it’s transformed me, and even though I’ve come back I’m still nowhere near as sick as I was, and I still haven’t needed hospital.” - No age provided, female, member for 23 years.

Members described Stepping Stone Clubhouse’s accepting culture as one of the most important parts of their recovery journey - to be seen as more than their mental illness diagnosis. In contrast to other community settings where symptoms or limitations were sometimes *“tolerated”*, the Clubhouse was viewed as valuing and celebrating member’s authentic selves. Members also expressed a sense of belonging, confidence, and appreciation in knowing their skills were essential to completing the Work-Ordered Day tasks or facilitating other Stepping Stone Clubhouse programs. Some members even felt a sense of identity in being known for certain skills (e.g., *“I’ve become the swimming person”*).

In my life, I’ve never had people feel like I could do anything…I got asked by one of the staff if I wanted to get up and speak [for a presentation], and I’d never been asked before. I thought I wasn’t worthy of anything like that. I still talk about it because I was so ecstatic to have been asked.

– 63-year-old female, member for 17 years.

### Evaluative Capacity Building

Since the completion of this evaluation project, Stepping Stone Clubhouse has integrated ongoing data collection mechanisms at both Orientation and Annual Progress collections. This is an important step towards sustained evaluative practice and a direct outcome of the PAR approach taken in this project. By embedding data collection processes within routine activities, Stepping Stone Clubhouse can glean insights into member outcomes, supporting a culture of evidence-based decision-making and continuous quality improvement. Stepping Stone Clubhouse has also established a group of ‘Evaluation Champions’ (staff and members) who will maintain the evaluative practices as part of the Work-Ordered Day. These Evaluation Champions will also lead the ongoing refinement of future evaluation questions, as well as data cleaning and analysis processes (with support from the Evaluation Reference Group).

## Discussion

In this evaluation of Stepping Stone Clubhouse, it was found that: people who were existing members of the Stepping Stone Clubhouse (for between 11 months – 28 years) had more positive outcomes than new members who had just joined (0–14 days); patterns of engagement with Clubhouse activities are different between new and existing members; and that the impact of Stepping Stone Clubhouse is created through personal and social acceptance. This evidence adds to the extensive field of existing Clubhouse evaluations, as the first published evaluation of an Australian-based Clubhouse and for reporting on the PAR approach employed and the impacts of taking this approach.

The sample in this study had relatively high psychosocial recovery scores compared to other similar populations. Scanlan et al. (2018) found that in a group of adults diagnosed with schizophrenia, depression, anxiety, bipolar disorder, and personality disorder, their pre-service RAS-DS scores were 64% for Personal Recovery, 66% for Functional Recovery, 62% for Symptom Management Recovery, and 68% for Social Recovery (Scanlan, 1993). Our study sample, regardless of their length of membership, had higher recovery scores. This may be because the act of coming to the Clubhouse is indicative of a more progressed stage of recovery. With regards to distribution of recovery scores, new members had a wider range of scores (in comparison to existing members) for all recovery domains. This finding speaks to a more diverse range of experiences of recovery for new members compared to existing members who have been accessing services at the Clubhouse for longer. Notably, both existing and new members indicated that symptom management recovery was the lowest domain of recovery. This underscores the challenging and longitudinal nature of managing the negative effects of mental illness, which is attributed to the complexity and chronicity of diagnoses of members from the Clubhouse. Psychological factors and comorbidities further complicate the development of skills and strategies to manage these negative effects (or symptoms). While the Clubhouse services may not appear as influential in this domain, sustained recovery over time requires comprehensive emphasis and support for symptom management within and across services.

In our study sample, existing members exhibited social connectedness levels akin to those reported in literature on psychosocial rehabilitation programs, while new members initially resembled those in less socially interactive care facilities (Brekke & Aisley, 1990; Levin & Brekke, 1993). These previous publications reported social connectedness scores of 45.3 and 47.2 at psychosocial rehabilitative sites that relied on client-to-client interactions compared to 33.2 at a care facility that had less socialization focus and low interactive environment (Levin & Brekke, 1993). A recent review explored the social networks and social support of Clubhouse members and how this impacted their recovery (Meyer et al., 2023). This review found that perceived levels of support and reciprocity, greater family support, a positive relationship with one highly supportive person, and connection to more mental health professionals were all associated with higher recovery scores (measured through the RAS-DS). Future evaluations of Stepping Stone Clubhouse may need to consider a broader definition of social connectedness than is captured by the CIS tool to include concepts of perceived belonging, and social integration with members’ broader chosen communities.

The quality-of-life scores reported by new members in the current study were comparable to those reported in previous publications with similar psychosocial rehabilitation services, and existing members were slightly higher than previous reports. Previous studies have used the MANSA tool with similar populations (Gillard et al., 2022; Bayliss et al., 2019; Lange et al., 2022), and reported scores of 4.0 to 4.2 amongst participants of psychosocial rehabilitation services, and a score of 3.9 for participants of a usual care comparator group. Importantly, the items within the MANSA that were higher for existing members compared to new members were the domains that are closely targeted through the Clubhouse Model.

The existing members in our study sample had a much lower rate of hospitalization in the past six months than new members. This is consistent with previous research that shows the Clubhouse Model delays re-hospitalization and can reduce the likelihood of rehospitalization for people with a diagnosed mental illness (McKay et al., 2018). Our qualitative findings with existing members mirrored this, with many members reporting that early in their journey at Stepping Stone Clubhouse they were in and out of hospital and that over time, while they still experienced negative symptoms of their mental illness, they felt they could manage symptoms without hospitalization.

This evaluation showed that existing members had higher rates of employment than new members. This speaks to the Clubhouse Model supporting employment by providing specific employment services (such as, transitional employment) and supports (such as, individual & employment support). Transitional employment helps members identify their strengths and interests through vocational assessments. The Clubhouse Model also offers real-world work experiences within the Clubhouse environment as a starting point to progress towards transitional, supported, or independent employment. This structured approach to stepping into employment is evident in the forms of employment observed between existing and new members, enabling members to transition through different forms of employment. This is also reflected in the aspirations of both new and existing members, with a majority of both groups expressing a desire to participate in the Work-Ordered Day or attain some form of further employment.

Education is crucial for populations with mental illness diagnoses or ill-health as it encourages individuals to develop a sense of empowerment, reduces stigma, improves decision-making, increases employment opportunities, fosters social integration, provides cognitive stimulation, encourages advocacy, and contributes to an overall improved quality of life (Kondirolli & Sunder, 2022). In the current study, both new and existing members were comparable not only in their current qualifications, but also in wanting further education. This finding shows there is room to strengthen how these aspirations are supported at the Stepping Stone Clubhouse. There may be need to further foster educational support services, including assistance with enrolment, navigating academic requirements, and accessing resources.

In this evaluation, the new member sample was younger and less likely to be diagnosed with schizophrenia compared to the existing member sample. This represents an interesting trend for Stepping Stone, potentially attracting different kinds of people to join the Clubhouse compared to the historic reach of the service. Historically, Stepping Stone received funding from a Schizophrenia Fellowship. This means there is a strong presence of members living with schizophrenia, however this image of the traditional Stepping Stone member may be shifting as the new member data indicates a diagnosis profile more like that seen in the general adult population living with mental illness in Queensland, Australia (data not shown). These differences in diagnostic profiles could potentially influence the outcomes observed in the current study, warranting cautious interpretation and contextualization of findings within the specific demographic and diagnostic context of the sample. One consideration is the variability in the chronicity and nature of different mental illness diagnoses among Clubhouse members, with conditions such as schizophrenia often characterized by long-term symptom management needs, contrasting with anxiety disorders which may present differently in terms of symptom severity and chronicity. While this may be an important note for future evaluations, the diverse mental health journeys of different diagnoses and life circumstances are well-served by the flexibility of the Clubhouse Model. The variety of Clubhouse services allows people with differing needs to access services as needed, and unconditional membership provides the stability and acceptance needed to work towards recovery.

Reflecting on the evaluation processes employed in this study, several key observations emerge. Firstly, member engagement throughout the evaluation was notably strong, indicating a high level of interest and investment from Stepping Stone Clubhouse community in contributing to the evaluation. This robust member engagement not only facilitated data collection but also enriched the interpretation of data analysis through interactive group discussions with members, as part of the PAR approach. In addition, the Evaluation Reference Group proactively embedded reflective practices throughout the study which underscored a commitment to continuous improvement and responsiveness to emerging needs. Through this process, the Evaluation Reference Group identified areas for refinement and adaptation, leading to tangible changes in evaluation methodologies and approaches during the study period. Notably, the refinement of the logic model following this study reflects a proactive effort to align evaluation frameworks more closely with the evolving understanding of Clubhouse operations and member impacts. These practices, as part of the PAR approach, deeply strengthened the validity of the current evaluation and would be key recommendations for future Clubhouse evaluation practices.

### Limitations

This study was cross-sectional in design, meaning there was no capacity to detect improvements in outcomes within individuals over time. Given the different demographic profiles of the new members to the existing members, it is essential that longitudinal data is collected into the future. As noted, the Clubhouse has established ongoing evaluative practices to ensure this longitudinal comparison is possible. Furthermore, there was no non-member comparator group in the current evaluation design. Logistical challenges precluded the inclusion of a non-member comparator group in the current evaluation, however future evaluations could engage with either: individuals who tour Stepping Stone Clubhouse but do not become members; or with individuals who engage with a different service for mental health support.

The qualitative interviews in this evaluation uncovered themes of suicidal ideation and behavior among members, however these aspects of the member journey were not directly examined in the quantitative surveys. Given the significance of suicidal ideation, and that suicide prevention is part of the mission of the Clubhouse, there may be merit in exploring these outcomes more comprehensively in future evaluations to better understand their prevalence and impact within the Clubhouse community.

In the member surveys, hospitalizations were reported by members as: whether they had been to hospital for their mental health in the last 6 months, and the nature of the hospital occurrence (i.e., called emergency services, presented to emergency department and/or admitted to hospital). Thus, hospitalization data in this evaluation does not distinguish between ongoing treatment (e.g., visiting for monitored medication change or other hospital-based planned intervention) and presentations due to symptom acuity or crisis events.

Furthermore, it is important to acknowledge the composition of the current evaluation sample, which primarily consisted of regular attendees within the Clubhouse community. While this demographic representation enhances the internal validity of the study by capturing insights from individuals actively engaged with Clubhouse services, it may also introduce selection bias. The overrepresentation of participants who regularly attend the Clubhouse could potentially limit the generalizability of study findings to the broader population of individuals with mental illness diagnoses who may have varying levels of engagement with Clubhouse programs. Consequently, interpretation of the study outcomes should be tempered with an awareness of the sample’s demographic composition and its implications for the broader applicability of findings.

It is also important to note that future evaluations could consider more nuanced ways of categorizing Clubhouse members other than by duration of membership (e.g., new and existing). Our qualitative findings were clear that every person that comes to Stepping Stone Clubhouse has a different journey with their recovery and it is often not related to how long they have been attending the Clubhouse, so it may be important for members to self-identify the ‘stage’ of their journey they are at. Ongoing data collection at Stepping Stone will include an additional survey item, created by members that aims to identify the stage of the Clubhouse journey that a member is on.

## Conclusions

Overall, the findings from this evaluation of the Stepping Stone Clubhouse suggest that there is a positive impact of Clubhouse membership on individuals with a diagnosed mental illness or mental ill-health. Existing members exhibit more positive outcomes compared to new members. Moreover, the observed differences in patterns of engagement between new and existing members emphasize the evolving nature of Clubhouse participation over time and the importance of offering services that members engage with at their own discretion. Central to the Clubhouse Model’s efficacy is the creation of a supportive environment fostering personal and social acceptance. However, to comprehensively understand the long-term effects and trajectories of Clubhouse involvement, there is a critical need for longitudinal data collection (Mutschler, McShane, Liebman, & Group, 2024). By capturing data over extended periods, future studies can provide deeper insights into the impact of Clubhouse services on individuals’ mental health outcomes and overall wellbeing.

## Declarations

### Language Statement

We have used the term ‘mental illness’ throughout this article, as it is the term used to describe eligibility for membership to the International Clubhouse Model. However, we acknowledge that this is not always the language that people with lived experience prefer. During the project, we discussed with members their preferred language and used terminology like ‘mental health concerns’, ‘mental health diagnoses’ and ‘mental ill-health’.
